# Daniel Courgeau, *Understanding Human Life. A Methodological and Interdisciplinary Approach*. Methodos Series vol. 19., 2022, Springer, xv + 261 pp.

**DOI:** 10.1007/s10680-023-09673-4

**Published:** 2023-07-24

**Authors:** Jakub Bijak

**Affiliations:** University of Southampton, Southampton, UK

For the regular readers of *European Journal of Population*, Daniel Courgeau does not need introducing: A longstanding co-editor of the journal for over twelve years (1988–2001) and a prolific author and co-author of many books and articles spanning a wide range of topics, from pioneering work on event history analysis in demography, to more recent contributions on philosophy and methodology of social sciences. His last book, *Understanding Human Life*, published in Springer’s Methodos series, of which he is a joint editor (with Robert Franck) since 2002, brings together many of these threads and areas of interest in one rich volume.

The book is aimed at social and human scientists, with focus on demographers, but is written from a much broader, philosophical perspective. Following a brief introduction laying the ground for the ensuing discussion, the book consists of two main parts. Part I, entitled “How Certain Approaches May Lead to Misunderstanding Human Life”, forms a discursive polemic with two now largely discredited approaches to understanding, predicting, and engineering human fate—astrology and eugenics. Part II, on “What Can One Capture of a Human Life, and How?” makes a methodological attempt to map the current scientific knowledge—and its limits—concerning human life’s different dimensions.

The four chapters of Part I deal with the fundamental questions of human liberty and free will, determinism, and predictability of human life and the future more generally. Starting with historical and philosophical origins in Chapter 2, and finishing with a critical assessment of prediction of social and human phenomena in Chapter 5, Part I uses astrology and eugenics as running examples of powerful intellectual misconceptions. Astrology, discussed in more depth in Chapter 3, and contrasted with astronomy as its scientific counterpart, is shown to be at best full of spurious correlations. Eugenics, even disregarding its questionable moral aspects, is challenged in Chapter 4 on the grounds of its fundamental ecological fallacy and underlying statistical flaws, in contrast to the science of genetics. This part of the book has a strong pedagogical slant and value—it can make excellent reading and discussion material for postgraduate or even undergraduate courses in philosophy of science or research methods.

In Part II, Chapters 6 and 7 look at the role of stories and how they shape our understanding of human life. Chapter 6 examines imagined stories from the antiquity and middle ages, including the examples of the epic about Gilgamesh, the tragedy of Oedipus, and the medieval French romance of Henri de Joyeuse. Chapter 7 delves into different types of real biographies: from narrative to formalised, noting how the relevant stories and data have been socially constructed through the ages. For a contemporary academic reader, an account of Plato’s adventures in his quest for “policy impact” of his philosophy can also strike a familiar chord. This chapter is very rich in content and comprises two separate threads—the first one general, revolving around the notion of hermeneutics, and the second one more demographic—which could almost become two standalone chapters.

For demographers, the second part of Chapter 7 contains several illuminating insights and interpretations, based on the author’s account of the paradigmatic changes in demography (Courgeau & Franck, 2007). Following Adolphe Quetelet and Antoine Cournot, as well as the author’s earlier work (Courgeau, 2012), it is suggested that “no mathematical tool other than probability can turn social science into a science” (p. 152). The life course can be then seen as a realisation of a stochastic process with a long memory, but the tools commonly used to account for heterogeneity of human experience, such as frailty, can be very sensitive to their assumptions (pp. 165–166). Due to their innate differences, the hermeneutic and scientific approaches seem wholly incompatible.

The psychological Chapter 8 focuses at memory—both verbal and visual—and its impact on the social measurement of various facets and processes of life. A prominent example includes recall biases in surveys, which in many contexts can just introduce some ‘background noise’ to the data, affecting the exact timings, but crucially not the sequence of successive life-course events. A general methodological discussion in this chapter looks into the ‘replication crisis’ in psychology and the ‘theory crisis’ in demography (Burch, 2018).

Building on this, the final and most demanding Chapter 9 examines the causal mechanistic system-based approach to studying interactions between individuals, populations they belong to, and the environments they inhabit. Such methods as agent-based or system dynamics modelling are portrayed as offering a possible synthesis, with trade-offs between their levels of complexity and depth of description. They can also bridge the seemingly irreconcilable gap between the hermeneutic and scientific explanations, identified in Chapter 7. This chapter also includes a timely discussion about the limits of artificial intelligence algorithms and their disconnection from actual thinking and other neurobiological processes. One missed opportunity in this chapter is that the critique of some of the concepts and links, such as between human intelligence and the notion of abductive reasoning (Peirce 1931–1958), are not spelled out more fully.

Bringing some of the less well-known French-language literature in many areas, such as the mathematical viability theory in Chapter 9, to the attention of English-speaking readers is an additional strength of this book. With the literature base being so rich and diverse, it is all the more surprising that the publisher decided to split the bibliography by chapter as in an edited collection—given that this book is a monograph, cross-checking the cited works would be greatly helped by having all references in one place. Still, the English translation by Jonathan Mandelbaum is excellent, and for true linguistic geeks, the book is peppered with footnotes with original Greek, Latin, Hebrew, or French quotes.

Overall, *Understanding Human Life* is an accomplished book, different parts of which can serve many readers very well: From providing stimulating discussion material for students, through inspiring researchers to critically consider various methodological approaches to analysing human life, to situating many features of demographic and social science thinking in historical and philosophical context. This is not necessarily a book to break new grounds with innovative research findings. It is, however, an erudite, interdisciplinary, and pedagogical volume, well worth reading and thinking about. With its surprising insights, it offers a rare broad-picture perspective. This can only come from the scientific experience of someone who—like the author—was already at the forefront of demographic innovation throughout his career.
